# Induction of protective interferon-β responses in murine osteoblasts following *Staphylococcus aureus* infection

**DOI:** 10.3389/fmicb.2022.1066237

**Published:** 2022-12-02

**Authors:** M. Brittany Johnson, Kelli H. Furr, Samantha R. Suptela, Whitney Leach, Ian Marriott

**Affiliations:** ^1^Department of Biological Sciences, University of North Carolina at Charlotte, Charlotte, NC, United States; ^2^Department of Molecular Biology, Stowers Institute for Medical Research, Kansas City, MO, United States

**Keywords:** osteoblasts, *Staphylococcus aureus*, osteomyelitis, interferon-beta, interferon stimulated genes

## Abstract

**Introduction:**

The refractory and recurrent nature of chronic staphylococcal osteomyelitis may be due, at least in part, to the ability of *Staphylococcus aureus* to invade and persist within bone-forming osteoblasts. However, osteoblasts are now recognized to respond to *S*. *aureus* infection and produce numerous immune mediators and bone regulatory factors that can shape the host response. Type I interferons (IFNs) are best known for their antiviral effects, but it is becoming apparent that they impact host susceptibility to a wide range of pathogens including *S*. *aureus*.

**Methods:**

Here, we have assessed the local expression of IFN-β by specific capture ELISA in an established in vivo mouse model of staphylococcal osteomyelitis. RNA Tag-Seq analysis, specific capture ELISAs, and/or immunoblot analyses, were then used to assess the expression of type I IFNs and select IFN stimulated genes (ISGs) in *S*. *aureus* infected primary murine osteoblasts. The effect of IFN-β on intracellular *S*. *aureus* burden was assessed in vitro following recombinant cytokine treatment by serial colony counts of liberated bacteria.

**Results:**

We report the presence of markedly elevated IFN-β levels in infected bone tissue in a mouse model of staphylococcal osteomyelitis. RNA Tag-Seq analysis of *S*. *aureus* infected osteoblasts showed enrichment of genes associated with type I IFN signaling and ISGs, and elevated expression of mRNA encoding IFN-β and ISG products. IFN-β production was confirmed with the demonstration that *S*. *aureus* induces its rapid and robust release by osteoblasts in a dose-dependent manner. Furthermore, we showed increased protein expression of the ISG products IFIT1 and IFIT3 by infected osteoblasts and demonstrate that this occurs secondary to the release of IFN-β by these cells. Finally, we have determined that exposure of *S*. *aureus*-infected osteoblasts to IFN-β markedly reduces the number of viable bacteria harbored by these cells.

**Discussion:**

Together, these findings indicate an ability of osteoblasts to respond to bacteria by producing IFN-β that can act in an autocrine and/or paracrine manner to elicit ISG expression and mitigate *S*. *aureus* infection.

## Introduction

*Staphylococcus aureus* is the most common causative agent of osteomyelitis and this condition is associated with severe inflammation and progressive bone loss. Staphylococcal osteomyelitis is often refractory to current treatment strategies and can manifest as recurrent infections (Funk and Copley, 2017; Ben-Zvi et al., 2019; Urish and Cassat, 2020). This may be due, at least in part, to the ability of *S*. *aureus* to invade and persist within bone cells (Jevon et al., 1999; Ellington et al., 2003, 2006; Mohamed et al., 2014; Strobel et al., 2016). *S*. *aureus* sequestered in the cytoplasm of bone-forming osteoblasts could provide a reservoir of bacteria and contribute to recurrent chronic staphylococcal osteomyelitis that occurs despite the presence of antibiotics and a seemingly adequate humoral response (Ellington et al., 2003, 2006; Mohamed et al., 2014). Importantly, osteoblasts are now recognized to express an array of pattern recognition receptors (PRRs), including cytosolic sensors, that enable them to respond to bacterial motifs (Gasper et al., 2002; Madrazo et al., 2003; Zou et al., 2003; Marriott et al., 2005; Chauhan and Marriott, 2010) and produce immune mediators and factors that regulate bone-resorbing osteoclast formation and activity (Madrazo et al., 2003, 2004; Marriott et al., 2004, 2005). As such, it is becoming evident that infected osteoblasts play a critical role in shaping the host response to *S*. *aureus* and the abnormal bone remodeling associated with staphylococcal infections (Marriott, 2004).

The type I interferons (IFNs), such as IFN-β, are best known for their antiviral effects, but it is becoming increasingly apparent that they can impact host susceptibility to a wide range of pathogens including bacteria (Peignier and Parker, 2021). However, these cytokines appear to exert variable effects in this regard and can be either protective or deleterious. Indeed, type I IFNs have been reported to have dual effects on *S*. *aureus* infections, with IFN-α/β receptor (IFNAR) deficient mice showing decreased mortality in a staphylococcal pneumonia model (Martin et al., 2009; Parker and Prince, 2012; Parker et al., 2014), while an anti-IFNAR1 subunit antibody has been reported to increase lung *S*. *aureus* burden (Spolski et al., 2019). Interestingly, a murine osteoblast-like cell line has been shown to express mRNA encoding IFN-β in response to polyinosinic:polycytidylic acid (Poly(I:C)), consistent with the anti-viral functions of this cytokine, and release of this protein was inferred from the ability of an IFN-β neutralizing antibody to partially inhibit osteoblast responses to this Toll like receptor 3 (TLR3) agonist (Nakamura et al., 2007). More recently, constitutive IFN-β production has been reported by osteocytes in a 3D *in vitro* co-culture system (Hayashida et al., 2014). However, the ability of osteoblasts to produce or respond to type I IFNs following *S*. *aureus* infection has not been investigated.

In the present study, we report markedly elevated IFN-β levels in infected bone tissue in a mouse model of post-traumatic staphylococcal osteomyelitis. RNA Tag-Seq analysis of *S*. *aureus* infected murine osteoblasts showed enrichment of genes associated with type I IFN signaling and IFN stimulated genes (ISGs), and elevated expression of mRNA encoding IFN-β and ISGs including IFIT1 and IFIT3. IFN-β production was confirmed with the demonstration that *S*. *aureus* induces its rapid and robust release by osteoblasts in a dose-dependent manner. Furthermore, we have demonstrated the increased expression of IFIT1 and IFIT3 proteins by infected osteoblasts and showed that such inductions occur secondary to the release of IFN-β by these cells. Finally, we have determined that exposure of *S*. *aureus*-infected osteoblasts to IFN-β can markedly reduce the number of viable bacteria harbored by these cells. Together, these findings indicate an ability of osteoblasts to respond to *S*. *aureus* challenge by producing IFN-β that can act in an autocrine and/or paracrine manner to elicit ISG expression and limit intracellular bacterial burden.

## Materials and methods

### *Staphylococcus aureus* propagation

*Staphylococcus aureus* strain UAMS-1 was grown on Luria broth (LB) agar plates overnight at 37°C in 5% CO_2_ and was cultured in LB on an orbital rocker at 37°C in 5% CO_2_ overnight. Prior to use in our *in vivo* and *in vitro* studies, bacteria were grown to mid-log phase in LB at 37°C with 5% CO_2_ and the number of colony forming units (CFU) was determined by spectrophotometry using a Genespec3 spectrophotometer (MiraiBio Inc.).

### Murine model of *Staphylococcus aureus* osteomyelitis

In these studies, we have used a murine model of *S*. *aureus* osteomyelitis, previously employed by our laboratory (Marriott et al., 2004, 2005; Chauhan and Marriott, 2010), that reproduces the pathology of human post-traumatic osteomyelitis (Spagnolo et al., 1993). Mice were anesthetized with inhalant isoflurane and the left femur was surgically exposed and abraded using a high-speed drill with a burred bit. Femurs were inoculated with *S*. *aureus* containing agarose beads, a method that induces localized infection rather than establishing a systemic bacterial infection. Agarose beads containing *S*. *aureus* were prepared by combining 1.4% low melt agarose cooled to 40–42°C with 1 × 10^9^ CFU *S*. *aureus*, followed by mineral oil addition with vigorous shaking on ice. Agarose beads were washed, stored on ice, and then administered to the abraded left femurs. Following application, the muscle fascia was sutured and the surgical incision closed with staples. At 3 days following *S*. *aureus* challenge, the infected left femur and contralateral uninfected femurs were isolated, weighed, and total protein extracted using T-PER. The protein concentration of IFN-β was evaluated by specific capture ELISA and normalized to femur weight.

### Isolation and culture of primary murine osteoblasts

Whole calvaria were isolated from two to three-day old murine neonates essentially as we have described (Bost et al., 2001; Gasper et al., 2002; Madrazo et al., 2003) with the following modifications. Briefly, primary osteoblasts were isolated from whole calvaria through six sequential 15-min trypsin/collagenase P digestions and the cells were maintained in DMEM supplement with 10% FBS and 1% penicillin/streptomycin at 37°C in a 5% CO_2_ atmosphere. At 24 h, primary osteoblasts were plated in 6-well plates at a density of 2 × 10^5^ cells per well and differentiated in αMEM supplemented with 10% FBS, 0.1 M ascorbic acid, 1 M β-glycerol phosphate, and 100 U/mL penicillin/100 μg/mL streptomycin at 37°C in a 5% CO_2_ atmosphere. The differentiation media was changed every other day until the experiment/infection at day 10, and the presence of mature osteoblasts was confirmed using an alkaline phosphatase staining kit (Abcam) and positive staining assessed by light field microscopy as we have described (Johnson et al., 2022).

### *In vitro Staphylococcus aureus* infection of osteoblasts

Cells were infected with *S*. *aureus* at multiplicities of infection (MOI) between 50:1 and 150:1 bacteria to each bone cell, as indicated, in antibiotic-free media at 37°C with 5% CO_2_ for 2 h, and then the media was replaced with media containing 100 U/mL penicillin/100 μg/mL streptomycin to kill residual extracellular bacteria for the duration of the experiment. At the indicated time points following bacterial infection, cell supernatants and whole cell protein isolates were collected. In studies evaluating intracellular *S*. *aureus* viability, infected osteoblasts (2 × 10^6^ cells) were lysed using 1% saponin in antibiotic-free media and the number of liberated viable bacteria was determined by serial colony counts.

### RNA sequence analysis

Primary murine osteoblasts were infected with *S*. *aureus* at a bacteria-to-osteoblast ratio of 75:1 and RNA was isolated using a PureLink RNA mini kit at 4 h post-infection. The Genomic Sequencing and Analysis Facility at the University of Texas at Austin prepared Tag-Seq libraries using an established method (Meyer et al., 2011) and sequenced libraries using an Illumina HiSeq 3,000. The 100 bp, single-end reads were trimmed and quality-filtered using the FastX-toolkit (Pearson et al., 1997). The trimmed SOLiD reads were mapped to the reference genome (GRCm38.p6) using BIOSCOPE V. 1.3.1 software. Differential expression analysis was performed using R package DESeq2 (Love et al., 2014). Outlier analysis was performed using the ArrayQualityMetrics R package (Kauffmann et al., 2009). Normalized and rlog-transformed counts were used in all downstream analysis (_R_3.5.0, R Core Team, 2015), following generation of a read-counts-per-gene file retaining only transcripts mapped to a single gene (included as supplemental information for (Sipprell et al., n.d.)). Gene expression heatmaps were made using the PHEATMAP package clustering expression patterns hierarchically (Wickham, 2009). A log_2_ ratio of read counts above 0.5 was considered statistically significant (padj <0.1). Pairwise comparisons between each treatment at each timepoint using Wald tests in DESeq identified significantly differentially expressed genes (DEGs) with *p*-values calculated using the Benjamini-Hochberg procedure. False discovery rate (FDR) adjustment for multiple testing resulted in 606 DEGs. Of the 606 differentially expressed genes identified, 135 genes displayed greater than a 2-fold difference in expression. Genes encoding type I IFN signaling components and IFN-stimulated genes (ISGs) were identified *via* gene ontology and Kyoto Encyclopedia of Genes and Genomes (KEGG) pathway analysis conducted using ShinyGO 0.76. For this analysis, all differentially expressed genes were converted to ENSEMBL gene IDS or STRING-db protein IDs. An FDR cutoff value of 0.05 and a pathway size range of 2 to 2000 was applied. The top 10 pathways were first selected by FDR and then sorted by fold enrichment. The original datasets are available in the Gene Expression Omnibus (GEO) publicly accessible repository under the accession number GSE 217455.

### Enzyme-linked immunosorbent assays

Specific capture ELISAs were conducted to quantify cell release of IFN-β and production of IFIT3 in response to *S*. *aureus* infection. A polyclonal goat anti-mouse IFN-β capture antibody (BioLegend, Cat # 519202, Clone Poly5192; 1 μg/mL) and an Armenian hamster biotin anti-mouse IFN-β detection antibody (BioLegend, Cat # 508105, Clone MIB-5E9.1; 2 μg/mL), were used to quantify mouse IFN-β concentrations. Streptavidin-HRP (R&D Systems; Lot # P198628) was added prior to the addition of tetramethylbenzidine substrate. The colorimetric reaction was stopped with 1:30 H_2_SO_4_ and the absorbance was measured at 450 nm. Recombinant murine IFN-β (BioLegend) was utilized to generate standard curves and extrapolation of the absorbance to the standard curve was used to determine the concentration of IFN-β in the cell supernatants. IFIT3 production was analyzed using a commercially available kit (MyBiosource) according to manufacturer guidelines.

### Immunoblot analyses

Total cell lysates were evaluated by immunoblot analysis for the presence of IFIT1, IFIT3, and PLSCR1. Immunoblots were incubated with a monoclonal antibody directed against IFIT1 (Novus Biologicals, clone OTI3G8), a polyclonal antibody directed against IFIT3 (Invitrogen), or a monoclonal antibody directed against PLSCR1 (Invitrogen, clone ARC2028), overnight at 4°C. Immunoblots were then washed and incubated with a horseradish peroxidase (HRP)-conjugated anti-mouse or anti-rabbit secondary antibody, and bound antibody was detected with a Pierce enhanced chemiluminescence (ECL) immunoblotting substrate (ThermoFisher Scientific). Immunoblots were re-probed with a mouse monoclonal antibody against β-actin (Abcam, Cat # 49900; 0.13 μg/mL) to assess total protein loading. The immunoblots shown are representative of at least three separate experiments. Densitometric analysis was conducted using ImageLab software (BioRad) and IFIT1, IFIT3, and PLSCR1 protein levels were normalized to β-actin expression.

### Statistical analysis

Data is expressed as the mean ± standard error of the mean (SEM). Commercially available software (GraphPad Prism, GraphPad Software, La Jolla, CA, United States) was used to conduct statistical analyses including Student’s *t* test, one-way analysis of variance (ANOVA) with Dunnet’s *post hoc* test, or two-way ANOVA with Tukey’s *post hoc* test as appropriate. In our RNA Tag-Seq analyses, differential expression analysis was performed using R package DESeq2. A log_2_ ratio of read counts above 0.5 was considered statistically significant (padj <0.1). Gene ontology analysis was conducted using ShinyGO 0.76. A false discovery rate (FDR) cutoff value of 0.05 and a pathway size range of 2 to 2000 was applied. The top 10 pathways were first selected by FDR and then sorted by fold enrichment. FDR was calculated based on nominal *p*-value from the hypergeometric test. Fold enrichment was defined as the percentage of genes in our list belonging to a pathway divided by the corresponding percentage in the background. In some analyses, Chi-squared and Student’s *t*-tests were used to determine whether differentially expressed genes have special characteristics when compared with genes in the reference genome. For all experiments, a value of p of less than 0.05 was considered statistically significant.

## Results

### *Staphylococcus aureus* infection elicits elevated local levels of IFN-β in a mouse model of osteomyelitis

**Figure 1:**
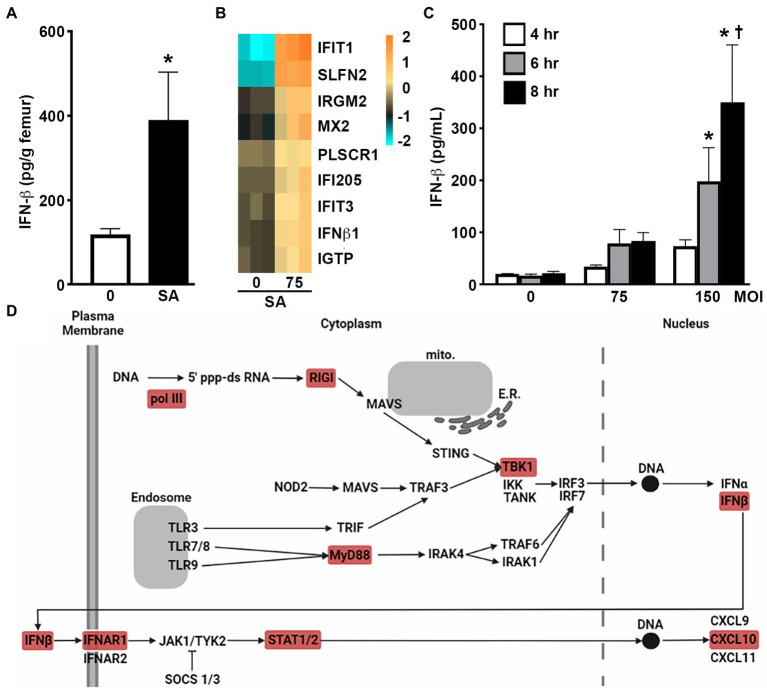
Murine osteoblasts produce IFN-β in response to *S*. *aureus* infection. **(A)** IFN-β levels in infected femurs (SA) and uninfected contralateral femurs (0) in a mouse model of staphylococcal osteomyelitis at 3 days post infection. Levels were determined by specific capture ELISA, normalized to bone weight, and shown as the mean ± SEM. Asterisk indicates a statistically significant difference from the uninfected femur (*p* < 0.05; *n* = 6). **(B,C)** Primary murine osteoblasts were uninfected (0) or *S*. *aureus* (SA) challenged (MOI of 75 or 150:1). At 4 h, mRNA expression was assessed by RNA Tag-Seq analysis and shown as a heatmap **(B)**, and IFN-β protein release was assessed at 4, 6, and 8 h by specific capture ELISA **(C)**. Data is shown as mean ± SEM (*n* = 9). Asterisks indicate a statistically significant difference from time-matched uninfected cells and daggers indicate a significant difference from cells at 4 h for that treatment. **(D)** Upregulation of genes associated with PRR and type I IFN signaling adapted from KEGG pathway analysis of RNA Tag-Seq data conducted using ShinyGO 0.76. Differentially expressed genes in these pathways are indicated in red.

To begin to assess the ability of bone cells to produce type I IFNs at sites of *S*. *aureus* infection, we have employed an established mouse model that reproduces post-traumatic staphylococcal osteomyelitis (Spagnolo et al., 1993; Marriott et al., 2004, 2005) and assessed the expression of IFN-β in infected femur bone tissue. As shown in Figure 1A. *S*. *aureus* infection elicited a marked increase in the level of IFN-β in bone tissue 3 days post-infection in comparison to contralateral uninfected control bone tissue.

### *Staphylococcus aureus* induces the expression of IFN-β by isolated primary murine osteoblasts

To determine the contribution made by resident bone cells to IFN-β production, we first assessed the changes in mRNA expression by primary murine osteoblasts following *S*. *aureus* challenge *via* RNA Tag-Seq analysis. Our recent gene ontology analysis demonstrated that osteoblasts displayed enrichment of gene products associated with type I IFNs, ISGs, and/or IFN-mediated cellular effects, following *S*. *aureus* challenge (Sipprell et al., n.d.). Specifically, osteoblasts showed a marked increase in the expression of mRNA encoding IFN-β as early as 4 h post infection (Figure 1B). Importantly, we have confirmed that such upregulation results in type I IFN protein production with the demonstration that *S*. *aureus* challenge elicits the rapid release of IFN-β by osteoblasts and does so in a dose-dependent manner (Figure 1C). Furthermore, KEGG pathway analysis has indicated the upregulated expression of mRNA encoding microbial pattern recognition receptor (PRR) components such as RNA polymerase III, retinoic acid inducible gene-I (RIG-I), myeloid differentiation primary response 88 (MyD88), and TANK-binding kinase 1 (TBK1), and IFN signaling components including IFNAR1 and STAT1/2 (Figure 1D).

### Murine osteoblasts express IFN stimulated genes in response to *Staphylococcus aureus* infection

**Figure 2:**
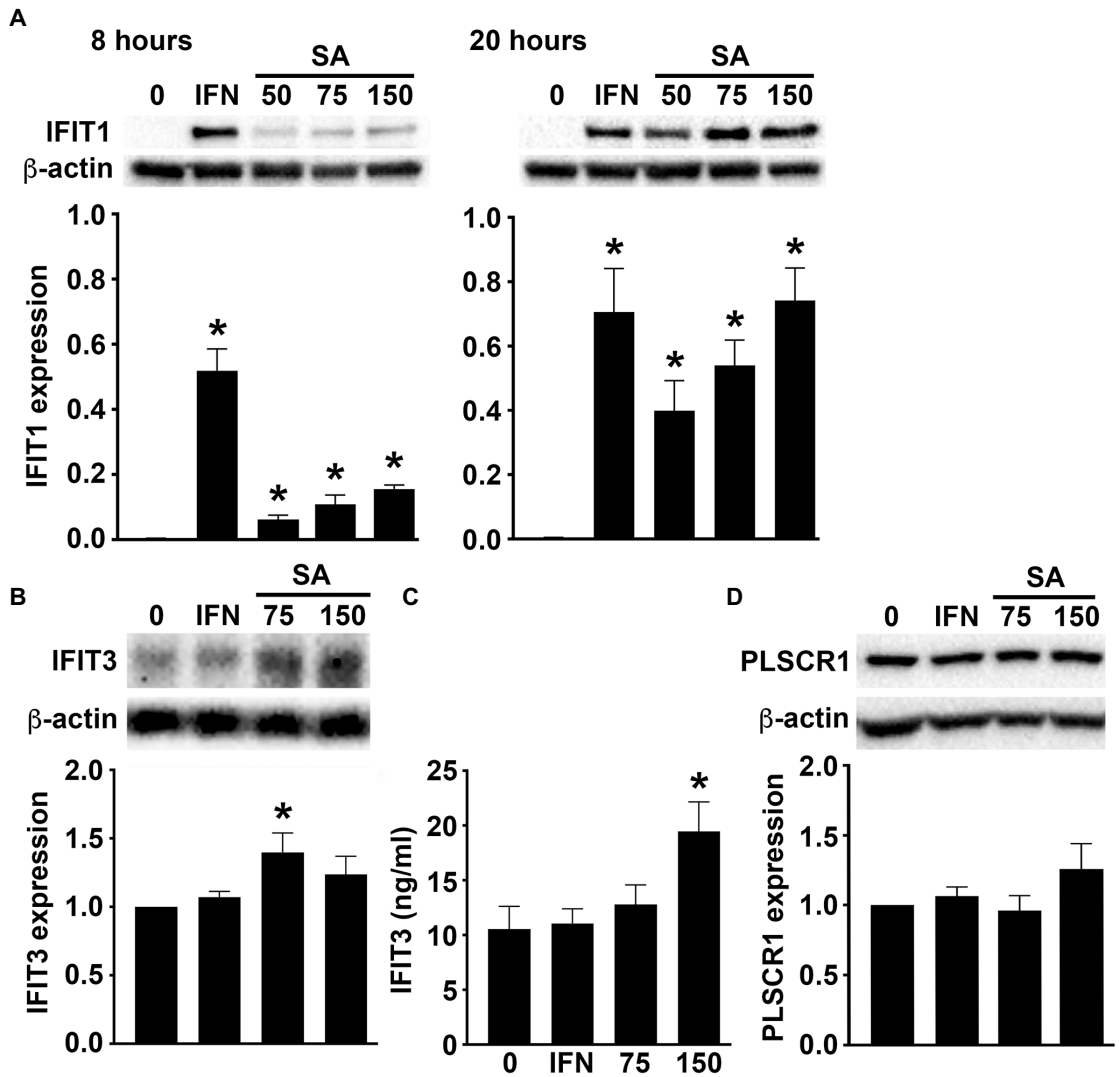
Murine osteoblasts express ISGs in response to *S. aureus*. Osteoblasts were uninfected (0) or *S*. *aureus* challenged (25, 50, 75, or 150:1). **(A)** At 8 (left) and 20 (right) hours, IFIT1 expression was determined by immunoblot analysis. As a positive control, cells were treated with recombinant IFN-β (1 ng/mL). A representative immunoblot is shown and average band intensities were determined and normalized to β-actin expression (*n* = 3). Asterisks indicate a statistically significant difference from uninfected/untreated cells (*p* < 0.05). **(B,C)** At 8 h, IFIT3 levels were determined by immunoblot analysis **(B)** and specific capture ELISA **(C)**. A representative immunoblot is shown and average band intensities were determined and normalized to β-actin (*n* = 6–9), and ELISA data is shown as mean ± SEM (*n* = 4). Asterisks indicate a statistically significant difference from uninfected cells (*p* < 0.05). **(D)** At 8 h, PLSCR1 expression was determined by immunoblot analysis. For comparison purposes, expression in osteoblasts treated with recombinant IFN-β (1 ng/mL) is shown. A representative immunoblot is shown and average band intensities were determined and normalized to β-actin (*n* = 5–11).

Our RNA Taq-Seq analysis also indicated the upregulated expression of an array of ISGs in primary murine osteoblasts following S. *aureus* infection. Specifically, osteoblasts showed marked increases in the expression of mRNA encoding IFIT1, IFIT3, SLFN2, IRGM2, MX2, PLSCR1, IFI205, and IGTP, as early as 4 h post-infection (Figure 1B). We then determined whether such upregulation results in increased ISG protein product levels in infected osteoblasts. As shown in Figure 2A *S*. *aureus* challenge induced the production of IFIT1 by osteoblasts in a time and dose-dependent manner. Similarly, infection elicited an increase in IFIT3 protein expression by osteoblasts over constitutive levels as determined by immunoblot analysis (Figure 2B) and specific capture ELISA (Figure 2C). In contrast, *S*. *aureus* infection failed to elevate PLSCR1 expression in osteoblasts above the robust levels seen in uninfected cells (Figure 2D) despite an apparent increase in mRNA expression encoding this ISG product (Figure 1B).

**Figure 3:**
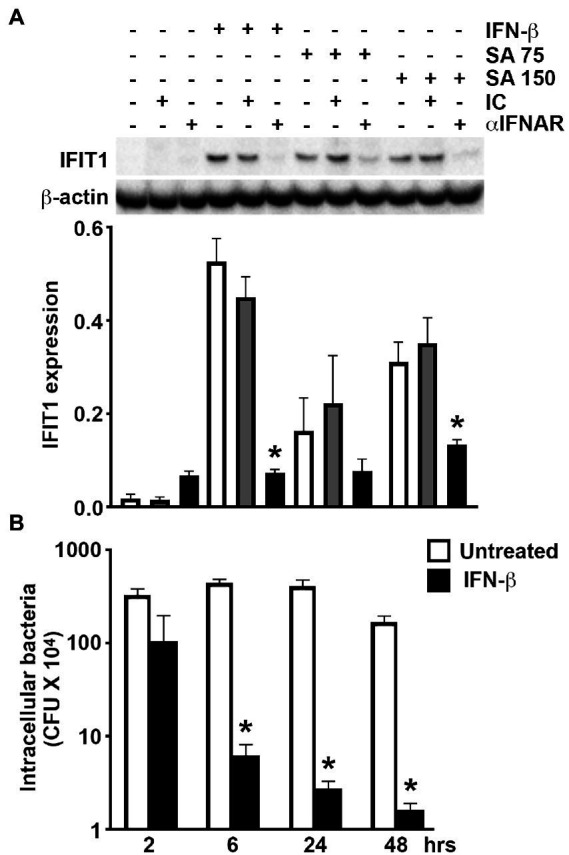
Osteoblast-derived IFN-β mediates ISG expression following *S*. *aureus* challenge and IFN-β can limit bacterial burden in infected cells. **(A)** Osteoblasts were untreated/uninfected, treated with recombinant IFN-β (1 ng/mL), or *S*. *aureus* challenged at MOI of 75 and 150:1 (SA 75 and SA 150, respectively), in the presence or absence of IFNAR blocking antibody (αIFNAR) or isotype control antibody (IC). At 8 h, protein isolates were taken and IFIT1 expression determined by immunoblot analysis. A representative immunoblot is shown and average band intensities were determined and normalized to β-actin expression (*n* = 4). Data is shown as the mean ± SEM (*n* = 4) and asterisks indicate a statistically significant difference from similarly stimulated cells treated with isotype control antibody (*p* < 0.05). **(B)** Osteoblasts (2 × 10^6^ cells) were infected with *S*. *aureus* (MOI of 75:1) in the absence or presence of recombinant IFN-β (1 ng/mL). At 2, 6, 24, and 48-h following infection, cells were lysed in antibiotic-free media and the number of viable intracellular bacteria were determined by colony count. Data is shown as the mean ±SEM (*n* = 6) and asterisks indicate a statistically significant difference from untreated cells (*p* < 0.05).

To determine whether *S*. *aureus* infection directly upregulates the expression of ISGs in osteoblasts or does so indirectly *via* the production and release of IFN-β, we have assessed the effect of IFNAR blockade on *S*. *aureus*-induced IFIT1 expression. As shown in Figure 3A, the presence of IFNAR blocking antibody significantly attenuated the increase in IFIT1 protein expression associated with *S*. *aureus* infection compared to infected osteoblasts exposed to an isotype control antibody. This data indicates that elevated ISG expression in *S*. *aureus* challenged osteoblasts occurs, in large part, to the actions of IFN-β produced by these cells.

### IFN-β can limit intracellular bacterial burden in *Staphylococcus aureus*-infected osteoblasts

The ability of osteoblasts to produce IFN-β in sufficient quantities to induce ISG expression in an autocrine and/or paracrine manner following bacterial challenge raises the possibility that such responses serve to limit *S*. *aureus* infections. To begin to test this hypothesis and assess the relevance of osteoblast type I IFN responses to this bacterium, we have determined whether IFN-β impacts the ability of *S*. *aureus* to persist/replicate intracellularly in these bone cells. As shown in Figure 3B, treatment of *S*. *aureus*-infected osteoblasts with recombinant IFN-β results in rapid and time-dependent reductions in the number of viable intracellular bacteria. Together, these results support the contention that osteoblasts produce IFN-β in response to *S*. *aureus* to induce antibacterial responses *via* IFNAR that limit intracellular bacterial burden.

## Discussion

It is becoming increasingly apparent that type I IFNs, including IFN-β, can impact host susceptibility to a wide range of pathogens including bacteria (Peignier and Parker, 2021). They have been reported to have both protective and deleterious effects on *S*. *aureus* infections, with decreased mortality observed with IFNAR deficient mice in a staphylococcal pneumonia model (Martin et al., 2009; Parker and Prince, 2012; Parker et al., 2014), while an anti-IFNAR1 subunit antibody has been reported to increase lung *S*. *aureus* burden (Spolski et al., 2019). Consistent with the notion that type I IFNs are produced in response to *S*. *aureus* infection and impact subsequent disease progression and severity, we have now demonstrated the local production of IFN-β in infected bone tissue in a mouse model of staphylococcal osteomyelitis.

Elevated IFN-β expression in *S*. *aureus* infected bone tissue may be attributable to production by infiltrating leukocytes. However, work from our laboratory and others have demonstrated that resident osteoblasts produce an array of products in response to *S*. *aureus* challenge that can regulate both bone homeostasis and inflammation (Marriott, 2004). Interestingly, a murine osteoblast-like cell line has been shown to express mRNA encoding IFN-β in response to the TLR3 agonist poly(I:C), and the ability of an IFN-β neutralizing antibody to partially inhibit their responses to this stimulus appears to support the ability of activated osteoblasts to release this cytokine (Nakamura et al., 2007). Similarly, transcriptome analysis of a human osteoblast-like cell line has indicated upregulated IFN-β mRNA expression during long-term (6-day) *S*. *aureus* infection (Nicolas et al., 2022). In addition, constitutive IFN-β production has recently been reported by mature osteoblasts/osteocytes in a 3D *in vitro* co-culture system (Hayashida et al., 2014). We have recently described the upregulated expression of 122 genes in primary murine osteoblasts following *S*. *aureus* challenge *via* RNA sequencing and gene ontology analysis (Sipprell et al., n.d.) and here we report that these genes include those encoding type I IFN proteins, IFNAR and associated signaling components, and ISGs. We show that these cells display increased expression of mRNA encoding IFN-β, IFNAR1, and STAT1/2, following infection (Figure 1B; Sipprell et al., n.d.). Importantly, we subsequently confirmed the significant release of IFN-β by infected osteoblasts and showed that it occurred in a bacterial dose-dependent manner. As such, these findings represent the first demonstration of *S*. *aureus*-induced production of IFN-β by osteoblasts.

While the mechanisms underlying IFN-β induction by this bacterium remain to be determined, internalized *S*. *aureus* can escape late endosomal/lysosomal compartment vesicles (Jauregui et al., 2013) to the cytoplasm *via* the production of cytotoxic phenol-soluble modulins (PSMs). There, they could be detected by cytosolic bacterial sensors such as nucleotide-binding oligomerization domain-containing protein 2 (NOD2), that we have shown to be functionally expressed by osteoblasts (Marriott et al., 2005; Chauhan and Marriott, 2010), which can trigger interferon regulatory factor (IRF) activation and initiate type I IFN gene expression (Mukherjee et al., 2019). Alternatively, *S*. *aureus* could induce IFN-β expression *via* cytosolic nucleic acid sensors and such a hypothesis is supported by the upregulated expression of cytosolic bacterial nucleic-sensing pathway components including RNA polymerase III, RIG-I, and TBK1, identified by KEGG pathway analysis of our recent RNA Taq-Seq analysis (Sipprell et al., n.d.).

The potential importance of osteoblast-mediated IFN-β production is underscored by the present demonstration that levels of mRNA encoding the ISGs, IFIT1, IFIT3, SLFN2, IRGM2, MX2, PLSCR1, IFI205, and IGTP, are markedly elevated in these cells following *S*. *aureus* challenge due, in large part, to autocrine and/or paracrine IFN-β signaling *via* IFNAR. While IFIT1 is an RNA-binding protein that can act as a sensor of viral single-stranded RNAs and inhibit viral mRNA expression, it has also been suggested to function as a negative regulator of inflammatory mediator production and a defective IFIT1-dependent IFN response has been shown to increase susceptibility to intracellular bacterial infection (John et al., 2018). Similarly, other ISGs, including SLFN2, have been reported to be protective against bacterial infection (as discussed in (Mavrommatis et al., 2013)). As such, ISG upregulation could serve to limit intracellular bacterial burden and this possibility is supported by the present demonstration that exposure of *S*. *aureus* infected osteoblasts to exogenous IFN-β markedly attenuates the number of viable bacteria harbored within these cells.

Interestingly, IFN-β can also have major effects on bone cell formation and/or function (Amarasekara et al., 2018, 2021). IFN-β is a potent inhibitor of osteoclastogenesis (Amarasekara et al., 2018) and has been reported to inhibit RANKL-induced osteoclastogenesis *via* a reduction in the expression of the critical signal transduction component c-Fos (Takayanagi et al., 2005; Avnet et al., 2007; Ha et al., 2008). This suggests that type I IFNs may serve as a negative feedback inhibitor of RANKL-induced osteoclastogenesis (Takayanagi et al., 2005). In addition, some evidence indicates that IFN-β might also inhibit osteoclast formation by enhancing NO production and iNOS signaling (Ha et al., 2008). However, ascribing a bone-preserving anti-osteoclastogenic role to type I IFNs is complicated by the fact that IFN-β can directly augment pro-osteoclastogenic inflammatory cytokine production. In addition, IFN-β has also been reported to be anti-osteoblastogenic (Zheng et al., 2006), and can inhibit bone formation and matrix mineralization by reducing the expression of multiple matrix components by osteoblasts (Woeckel et al., 2012; Deng et al., 2020). Furthermore, the finding that RANKL deficient mice show increased susceptibility to endotoxic shock while exogenous RANKL administration is protective (Maruyama et al., 2006), suggests that IFN-β-mediated decreases in RANKL production could exacerbate detrimental inflammation. As such, the net impact of type I IFN production by resident bone cells on the progressive inflammatory damage associated with staphylococcal osteomyelitis remains to be determined.

Collectively, the present study has identified a previously unknown type I IFN response by osteoblasts to *S*. *aureus* challenge, one that could have protective effects by limiting intracellular bacterial survival/propagation within these resident bone cells and has the potential to mitigate the pro-osteoclastogenic effects of mediators such as RANKL that are produced at sites of bone infection.

## Data availability statement

The datasets presented in this study can be found in online repositories. The name of the repository and accession number can be found at: NCBI Gene Expression Omnibus; GSE217455.

## Ethics statement

All protocols involving animals were approved by the Institutional Animal Care and Use Committee of the University of North Carolina at Charlotte.

## Author contributions

MJ, KF, and SS harvested primary murine osteoblasts, performed *in vivo* bone infections in mice, in accordance with approved IACUC protocols, prepared bacterial stocks, carried out the *in vitro* experiments, specific capture ELISAs, immunoblot analyses, and performed data analysis. WL assisted in the assessment and interpretation of the Tag-Seq RNA sequence analysis data. IM conceived the study, contributed to the experimental design, and drafted the manuscript. All authors read and approved the final version of the manuscript.

## Funding

This work was supported by grant AR074102 to IM from the National Institutes of Health.

## Conflict of interest

The authors declare that the research was conducted in the absence of any commercial or financial relationships that could be construed as a potential conflict of interest.

## Publisher’s note

All claims expressed in this article are solely those of the authors and do not necessarily represent those of their affiliated organizations, or those of the publisher, the editors and the reviewers. Any product that may be evaluated in this article, or claim that may be made by its manufacturer, is not guaranteed or endorsed by the publisher.
